# Food Intolerances and Food Allergies: Novel Aspects in a Changing World

**DOI:** 10.3390/nu17091526

**Published:** 2025-04-30

**Authors:** Aurelio Seidita, Stella Compagnoni, Mirco Pistone, Antonio Carroccio

**Affiliations:** 1 Internal Medicine Unit, “V. Cervello” Hospital, Ospedali Riuniti “Villa Sofia-Cervello”, Via Trabucco, 180, 90146 Palermo, Italy; aurelio.seidita@unipa.it (A.S.); stella.compagnoni.2011@gmail.com (S.C.); mirco.pistone@gmail.com (M.P.); 2 Department of Health Promotion Sciences, Maternal and Infant Care, Internal Medicine and Medical Specialties (PROMISE), University of Palermo, Piazza delle Cliniche, 2, 90127 Palermo, Italy; 3 Institute for Biomedical Research and Innovation (IRIB), National Research Council (CNR), 90146 Palermo, Italy

Despite the rapid progress that has considerably affected most fields of medicine in recent years, many gray areas still exist in our understanding of allergies/hypersensitivities and intolerances to foods. While these pathologies have been widely described and characterized from a clinical point of view, little is known about their pathogenesis, as their mechanisms are often unclear, especially when the clinical manifestations are predominantly gastroenterological [1].

The Food and Agriculture Organization and the World Health Organization, in an ad hoc expert consultation focusing on immunoglobulin E (IgE)-mediated reactions, have identified eight major categories of food allergens: eggs, milk, peanuts, sesame seeds, flour, fish, and shellfish [2,3]. The reactions to each allergen have different features, making the field of food allergies extremely varied: some, such as egg allergy, tend to improve with age, while others, i.e., fish and seafood allergies, tend to worsen, making their management extremely difficult [4]. The situation is even more complex in the case of hypersensitivities that are not triggered by an IgE-mediated mechanism.

Moreover, the objective difficulty of diagnosing and differentiating between food allergies/hypersensitivities and intolerances in some cases should be taken into account. Their clinical presentations are often heterogeneous, embracing a wide spectrum of manifestations: from simple dyspepsia to anaphylaxis and anaphylactic shock [2]. In this scenario, the diagnostic procedures available are often varied, not always standardized and sometimes very long, especially when an elimination diet and a subsequent (double) blind challenge is required [5]. The diagnostic difficulty is mainly linked to non-IgE-mediated reactions. In these cases, diagnostic armamentarium is limited, due to the uncertainties related to the pathogenetic mechanisms, thus making it particularly complex to identify effective and easily repeatable diagnostic biomarkers.

Therefore, the aim of this Special Issue is to focus on the field of food allergies/hypersensitivities/intolerances, exploring both the possible pathogenetic mechanisms underlying such conditions and existing management strategies, in the search for possible treatment perspectives.

## 1. Adherence and Quality of Life

A key point that should be stressed is the potential impact of food allergies/hypersensitivities/intolerances on the quality of life of affected subjects [6]. This issue has received increasing attention in the last 15 years following the emergence of non-celiac wheat sensitivity (NCWS), which seems to involve up to 5-6% of the general population. Cotton et al., applying scores such as the “Coeliac Dietary Adherence Test” (CDAT) to evaluate adherence to the diet and the “Coeliac Symptom Index” (CSI) and “Sleep Condition Indicator” (SCI) to analyze symptoms and sleep quality, studied this aspect by comparing patients with NCWS and celiac disease (CeD), and concluded that NCWS patients adhered less to the wheat-free diet (WFD) than CeD to a gluten-free diet (GFD) and this correlated with a worse quality of life and sleep [7].

However, adhering to a WFD/GFD is not always simple and immediate: involuntary exposure to gluten, high costs, and palatability are just some of the reasons that lead to poor compliance with these elimination diets [8,9,10]. Consequently, several strategies have been attempted to improve adherence in subjects with NCWS and other gluten-related disorders [11]. Among these, some study groups have focused on identifying probiotic blends that could be used to degrade gliadin, making gluten-free wheat flour more quickly available and affordable. If successful, this approach could represent a significant step towards improving the quality of life for the countless people around the world who struggle with gluten-related disorders. Following this line of research, results from Ramedani et al. showed that fermenting flour for 4 h with a probiotic mix composed of *B. longum*, *L. acidophilus*, and *L. plantarum* could represent a good therapeutic strategy [12].

## 2. Prevention and Development of Tolerance

New strategies for managing food hypersensitivities/intolerances involve their prevention, and can be introduced even during pregnancy and in the neonatal period [13]. A combination of maternal dietary interventions, breastfeeding, and the early introduction of (potential) food allergens to newborns might reduce the risk of food allergies in children, but further studies are underway to clarify how genetic, immunological, and environmental factors can interact in the pathogenesis of the pathology itself. In this scenario, Panagiotou et al. proved that the Mediterranean diet, rich in long-chain polyunsaturated fatty acids, could have beneficial effects to combat the development of food allergies and allergic symptoms in newborns [14]. Furthermore, during pregnancy and in the neonatal period, changes in the composition of the intestinal microbiota can play a key role in terms of creating either preemptive or risk factors for the development of food allergies/intolerances: the first two years of life, in particular, have proven to be crucial for exposure to risk or protective factors against the above-mentioned pathologies, with breastfeeding representing a factor promoting the formation of a “protective” microbiota, while the opposite is true for artificial formulas [15,16].

Another still very controversial and debated issue is the possible role of food processing methods, including the different types of cooking, on the allergenicity of some foods: It seems that some methods (e.g., heating, boiling, Maillard reaction, enzymatic cross-linking, and enzymatic degradation) can induce less significant reactions, even up to oral tolerance [17], but the underlying mechanisms have not yet been clarified and further studies are required to confirm such results.

## 3. Role of Histamine

Histamine is historically known to be one of the main mediators of hypersensitivity reactions. However, even though the physiopathology of the clinical manifestations related to histamine release is well understood, the reasons underlying the accumulation of histamine and the development of histaminergic syndromes of varying severity that affect predisposed subjects still need to be clarified.

The methods of cooking and preserving foods and, therefore, including lactic acid fermentation linked to bacterial degradation, might affect the amount of histamine present in a food [18]. Moreover, a reduced activity of diamine oxidase (DAO), expressed by intestinal epithelial cells, might also affect a food’s histamine levels, as occurs in histamine intolerance (HIT) [19]. Maintz et al. explored the possible diagnostic role of DAO, highlighting how this assay seems to be effective in discriminating between subjects with HIT and healthy subjects, but also underlining that, even today, the diagnosis remains predominantly clinical [20]. In this scenario, authors have shown how this condition is particularly marked in patients with pre-existing mucosal damage, such as those suffering from chronic inflammatory bowel disease or CeD, and the more severe the intestinal damage is, the greater the accumulation of histamine [21].

Even more difficult is the study of the pathogenesis and diagnosis of non-IgE-mediated hypersensitivities. Historical and new evidence has shown how the intestinal mucosa can react to foods with “histamine-like” hypersensitivity reactions and how these occur in many patients who have been previously classified as suffering from “simple” functional gastrointestinal disorders, such as irritable bowel syndrome or functional dyspepsia [22,23].

## 4. Lactose Intolerance and Other Milk-Related Food Hypersensitivities

Lactose intolerance is the most common adverse food reaction worldwide, with an estimated global prevalence of 57 to 65% [24]. This condition is often “self-diagnosed” by patients who, associating the onset of symptoms with the intake of milk and fresh dairy products, replace cow’s milk and its derivates with plant-based ones without seeking any medical or nutritional supervision [25]. However, subjects with “self-reported” milk intolerance appear to form a heterogeneous group [26]: in some of them, in fact, rather than a simple enzymatic deficiency, the pathogenic mechanism underlying the disorders seems to be of a hypersensitivity/inflammatory nature, a hypothesis corroborated by the high number of subjects with positive fecal calprotectin values (>50% in some studies) [27]. Such a high number of ‘self-diagnosed’ patients raises issues related to the adoption of elimination diets. Replacement of cow’s milk with plant-based ones could be harmful to these subjects due to the qualitative and quantitative diversity of their micro- and macronutrients. In fact, it has been shown that most plant-based beverages cannot fully replace cow’s milk in terms of nutritional quality [28,29]. Nevertheless, some authors analyzing the microbiological and chemical characteristics of plant-based milks have shown that these can represent a safe alternative to cow’s milk, while still requiring the pasteurization technique [30].

## 5. Precision Nutrition

Genetic diversities in specific individuals and ethnic groups can influence nutritional requirements, metabolism, and response to nutritional and dietary interventions [31]. For this reason, ever-increasing interest has been shown towards omics technologies (genomics, epigenomics, metabolomics, etc.), which, by shedding light on the complex interactions between the human body and nutrients, can help to diagnose and treat food allergy/hypersensitivity and intolerances [32,33], through what can be defined as “precision nutrition” [34]. The interpretation of omics data is very complex and easily misunderstood, so their analysis and correlation with symptoms must be performed by experts [33]. Nevertheless, this approach, although still at the dawn of its development, must be considered one of the most promising areas of development in the complex world of the diagnosis and management of allergies/hypersensitivity and food intolerances.

## 6. Therapeutic Approaches

To date, the treatment of food allergies/hypersensitivities/intolerances almost exclusively relies on the use of elimination diets, with the removal of one or more foods, often based on the patient’s self-perception [35].

Pharmacological therapy is only symptomatic and, at present, there are no drugs able to change the history of these diseases in the long term [35].

In this scenario, immunotherapy deserves a special mention. This approach is based on a gradual exposure to small and increasing doses of allergens, exploiting a suppression of the immune response mediated by food-specific IgE [36,37,38]. Currently, the most intense areas of immunotherapy research concern the oral administration route. Other possibilities are the subcutaneous and sublingual routes, which are used less frequently, especially due to the risk of severe adverse reactions in the former [39,40]. Desensitization procedures, far from completely healing patients, can only prevent the onset of severe symptoms in some food allergies, which, however, represent only a (small) part of the adverse reactions to foods.

## 7. Future Perspectives and Considerations

Food allergies/hypersensitivities/intolerances represent an increasingly significant emerging health problem, as does the globalized food market, which has increased exposure to new and potentially immunogenic foods (e.g., pre-prepared or containing flours or insect derivatives).

The complexity of the pathogenetic mechanisms involved in their development is the main limitation to finding the possible keystone for an effective therapeutic approach; thus, understanding these mechanisms represents the real challenge of future scientific research into this fascinating group of pathologies.

An increasing number of researchers are looking to omics sciences to define exactly how foods can trigger immune and non-immune reactions. Others, instead, are searching for answers to the unsolved questions in the mechanisms of food processing (e.g., preservation, cooking, etc.), or even analyzing the methods and timing with which the human body is exposed to the specific components of foods. Finally, several study groups are investigating the interaction between foods and the gut microbiota as the main culprit behind the development of food tolerance/sensitivity.

In conclusion, it is safe to state that the field of food allergies/hypersensitivities/intolerances requires further exploration, as it remains a part of medical science that is still obscure, due to uncertainties around the physiopathological mechanisms involved, which have repercussions both on the diagnosis, still today often based on poorly standardized and repeatable methods (e.g., double-blind placebo-controlled food challenges), and on the treatment, which almost exclusively relies on the use of elimination diets.

## Abbreviations

The following abbreviations are used in this manuscript:

CDAT	Celiac dietary adherence test
CeD	Celiac disease
CSI	Celiac symptom index
DAO	Diamine oxidase
GFD	Gluten-free diet
HIT	Histamine intolerance
IgE	Immunoglobulin E
NCWS	Non-celiac wheat sensitivity
SCI	Sleep condition indicator
WFD	Wheat-free diet

## References

[1] Sicherer S.H., Sampson H.A. (2018). Food Allergy: A Review and Update on Epidemiology, Pathogenesis, Diagnosis, Prevention, and Management. J. Allergy Clin. Immunol..

[2] Seth D., Poowutikul P., Pansare M., Kamat D. (2020). Food Allergy: A Review. Pediatr. Ann..

[3] Food and Agriculture Organization (FAO), World Health Organization (WHO) (2022). Risk Assessment of Food Allergens. Part 1: Review and Validation of Codex Alimentarius Priority Allergen List through Risk Assessment.

[4] Wai C.Y.Y., Leung N.Y.H., Leung A.S.Y., Wong G.W.K., Leung T.F. (2021). Seafood Allergy in Asia: Geographical Specificity and Beyond. Front. Allergy.

[5] Keet C.A. (2016). A Call to Improve Standards for Reporting of Diagnostic Test Research in Allergy. J. Allergy Clin. Immunol..

[6] Walkner M., Warren C., Gupta R.S. (2015). Quality of Life in Food Allergy Patients and Their Families. Pediatr. Clin. N. Am..

[7] Cotton C., Raju S.A., Ahmed H., Webster G., Hallam R., Croall I., Coleman S., Trott N., Rej A., Shiha M.G. (2023). Does a Gluten-Free Diet Improve Quality of Life and Sleep in Patients with Non-Coeliac Gluten/Wheat Sensitivity?. Nutrients.

[8] Versluis A., Knulst A.C., Kruizinga A.G., Michelsen A., Houben G.F., Baumert J.L., van Os-Medendorp H. (2015). Frequency, Severity and Causes of Unexpected Allergic Reactions to Food: A Systematic Literature Review. Clin. Exp. Allergy.

[9] Schiepatti A., Maimaris S., Nicolardi M.L., Alimenti E., Vernero M., Costetti M., Costa S., Biagi F. (2022). Determinants and Trends of Adherence to a Gluten-Free Diet in Adult Celiac Patients on a Long-Term Follow-up (2000–2020). Clin. Gastroenterol. Hepatol..

[10] Mazzola A.M., Zammarchi I., Valerii M.C., Spisni E., Saracino I.M., Lanzarotto F., Ricci C. (2024). Gluten-Free Diet and Other Celiac Disease Therapies: Current Understanding and Emerging Strategies. Nutrients.

[11] Dimidi E., Kabir B., Singh J., Ageridou A., Foster C., Ciclitira P., Dubois P., Whelan K. (2021). Predictors of Adherence to a Gluten-Free Diet in Celiac Disease: Do Knowledge, Attitudes, Experiences, Symptoms, and Quality of Life Play a Role?. Nutrition.

[12] Ramedani N., Seidita A., Asri N., Azimirad M., Yadegar A., Jahani-Sherafat S., Sharifan A., Mansueto P., Carroccio A., Rostami-Nejad M. (2023). The Gliadin Hydrolysis Capacity of *B. longum*, *L. acidophilus*, and *L. plantarum* and Their Protective Effects on Caco-2 Cells against Gliadin-Induced Inflammatory Responses. Nutrients.

[13] Manti S., Galletta F., Bencivenga C.L., Bettini I., Klain A., D’Addio E., Mori F., Licari A., Miraglia del Giudice M., Indolfi C. (2024). Food Allergy Risk: A Comprehensive Review of Maternal Interventions for Food Allergy Prevention. Nutrients.

[14] Panagiotou E., Andreou E., Nicolaou S.A. (2023). The Effect of Dietary Components of the Mediterranean Diet on Food Allergies: A Systematic Review. Nutrients.

[15] Wang S., Wei Y., Liu L., Li Z. (2022). Association Between Breastmilk Microbiota and Food Allergy in Infants. Front Cell Infect Microbiol.

[16] Notarbartolo V., Carta M., Accomando S., Giuffrè M. (2023). The First 1000 Days of Life: How Changes in the Microbiota Can Influence Food Allergy Onset in Children. Nutrients.

[17] Han X., Wang X., Chen X., Liu H., Liu J., Waye M.M.Y., Liu G., Rao S. (2024). Intervention Efficacy of Slightly Processed Allergen/Meat in Oral Immunotherapy for Seafood Allergy: A Systematic Review, Meta-Analysis, and Meta-Regression Analysis in Mouse Models and Clinical Patients. Nutrients.

[18] Smolinska S., Jutel M., Crameri R., O’Mahony L. (2014). Histamine and Gut Mucosal Immune Regulation. Allergy.

[19] Comas-Basté O., Sánchez-Pérez S., Veciana-Nogués M.T., Latorre-Moratalla M., Vidal-Carou M. (2020). del C. Histamine Intolerance: The Current State of the Art. Biomolecules.

[20] Maintz L., Novak N. (2007). Histamine and Histamine Intolerance. Am. J. Clin. Nutr..

[21] Dvornikova K.A., Platonova O.N., Bystrova E.Y. (2023). Inflammatory Bowel Disease: Crosstalk between Histamine, Immunity, and Disease. Int. J. Mol. Sci..

[22] Barbara G., Stanghellini V., De Giorgio R., Cremon C., Cottrell G.S., Santini D., Pasquinelli G., Morselli-Labate A.M., Grady E.F., Bunnett N.W. (2004). Activated Mast Cells in Proximity to Colonic Nerves Correlate with Abdominal Pain in Irritable Bowel Syndrome. Gastroenterology.

[23] Fritscher-Ravens A., Pflaum T., Mösinger M., Ruchay Z., Röcken C., Milla P.J., Das M., Böttner M., Wedel T., Schuppan D. (2019). Many Patients with Irritable Bowel Syndrome Have Atypical Food Allergies Not Associated with Immunoglobulin E. Gastroenterology.

[24] Catanzaro R., Sciuto M., Marotta F. (2021). Lactose Intolerance: An Update on Its Pathogenesis, Diagnosis, and Treatment. Nutr. Res..

[25] Paul A.A., Kumar S., Kumar V., Sharma R. (2020). Milk Analog: Plant Based Alternatives to Conventional Milk, Production, Potential and Health Concerns. Crit. Rev. Food Sci. Nutr..

[26] Carroccio A., Soresi M., Mantia B., Fayer F., La Blasca F., Seidita A., D’Alcamo A., Florena A., Tinè C., Garlisi C. (2021). Whole Cow’s Milk but Not Lactose Can Induce Symptoms in Patients with Self-Reported Milk Intolerance: Evidence of Cow’s Milk Sensitivity in Adults. Nutrients.

[27] Seidita A., Mansueto P., Giuliano A., Chiavetta M., Soresi M., Carroccio A. (2023). Fecal Calprotectin in Self-Reported Milk Intolerance: Not Only Lactose Intolerance. Nutrients.

[28] Fructuoso I., Romão B., Han H., Raposo A., Ariza-Montes A., Araya-Castillo L., Zandonadi R.P. (2021). An Overview on Nutritional Aspects of Plant-Based Beverages Used as Substitutes for Cow’s Milk. Nutrients.

[29] Jeske S., Zannini E., Arendt E.K. (2018). Past, Present and Future: The Strength of Plant-Based Dairy Substitutes Based on Gluten-Free Raw Materials. Food Res. Int..

[30] Giugliano R., Musolino N., Ciccotelli V., Ferraris C., Savio V., Vivaldi B., Ercolini C., Bianchi D.M., Decastelli L. (2023). Soy, Rice and Oat Drinks: Investigating Chemical and Biological Safety in Plant-Based Milk Alternatives. Nutrients.

[31] Ferguson L.R., De Caterina R., Görman U., Allayee H., Kohlmeier M., Prasad C., Choi M.S., Curi R., de Luis D.A., Gil Á. (2016). Guide and Position of the International Society of Nutrigenetics/Nutrigenomics on Personalised Nutrition: Part 1—Fields of Precision Nutrition. J. Nutr. Nutr..

[32] Pratelli G., Tamburini B., Badami G.D., Lo Pizzo M., De Blasio A., Carlisi D., Di Liberto D. (2024). Cow’s Milk: A Benefit for Human Health? Omics Tools and Precision Nutrition for Lactose Intolerance Management. Nutrients.

[33] Aruoma O.I., Hausman-Cohen S., Pizano J., Schmidt M.A., Minich D.M., Joffe Y., Brandhorst S., Evans S.J., Brady D.M. (2019). Personalized Nutrition: Translating the Science of NutriGenomics Into Practice: Proceedings From the 2018 American College of Nutrition Meeting. J. Am. Coll. Nutr..

[34] Marcum J.A. (2020). Nutrigenetics/Nutrigenomics, Personalized Nutrition, and Precision Healthcare. Curr. Nutr. Rep..

[35] Boyce J.A., Assa’ad A., Burks A.W., Jones S.M., Sampson H.A., Wood R.A., Plaut M., Cooper S.F., Fenton M.J., NIAID-Sponsored Expert Panel (2010). Guidelines for the Diagnosis and Management of Food Allergy in the United States: Report of the NIAID-Sponsored Expert Panel. J. Allergy Clin. Immunol..

[36] Damelio C.M. (2015). Successful Specific Oral Tolerance Induction with Hake in an Allergic Child Detecting Fish in Cooking Steam. J. Allergy Clin. Immunol..

[37] Nucera E., Ricci A.G., Rizzi A., Mezzacappa S., Rienzo A.D., Pecora V., Patriarca G., Buonomo A., Aruanno A., Schiavino D. (2018). Specific Oral Immunotherapy in Food Allergic Patients: Transient or Persistent Tolerance?. Adv. Dermatol. Allergol..

[38] Porcaro F., Caminiti L., Crisafulli G., Arasi S., Chiera F., La Monica G., Pajno G.B. (2016). Management of Food Allergy to Fish with Oral Immunotherapy: A Pediatric Case Report. Pediatr. Allergy Immunol. Pulmonol..

[39] Burks A.W., Sampson H.A., Plaut M., Lack G., Akdis C.A. (2018). Treatment for Food Allergy. J. Allergy Clin. Immunol..

[40] Chen M., Land M. (2017). The Current State of Food Allergy Therapeutics. Hum. Vaccin. Immunother..

